# Efficacy and safety of Qixue Tongzhi Granule in improving the exercise capacity of stable coronary artery disease: study protocol for a multicenter, randomized, double-blind, placebo-controlled trial

**DOI:** 10.3389/fcvm.2023.1186018

**Published:** 2023-08-17

**Authors:** Tiantian Chao, Jinghui Sun, Na Huan, Yaru Ge, Chenglong Wang

**Affiliations:** National Clinical Research Center for Chinese Medicine Cardiology, Xiyuan Hospital, China Academy of Chinese Medical Sciences, Beijing, China

**Keywords:** Qixue Tongzhi Granule, stable coronary artery disease, exercise capacity, randomized controlled trial, protocol

## Abstract

**Background:**

Despite optimal medical therapy, patients with stable coronary artery disease (SCAD) still have a high risk of recurrent cardiovascular events. Exercise capacity measured by cardiopulmonary exercise testing (CPET) is a good surrogate marker for the long-term prognosis of SCAD. Qixue Tongzhi Granule (QTG) is created by academician Chen Keji and has the function of tonifying qi, promoting blood circulation, and regulating qi-flowing. This trial aims to investigate the efficacy and safety of QTG in improving exercise tolerance, alleviating angina pectoris and anxiety/depression symptoms, promoting health-related quality of life, and reducing the risk of adverse cardiovascular events in subjects with SCAD.

**Methods:**

This is a randomized, double-blind, placebo-controlled trial. 150 SCAD patients with qi deficiency, blood stasis, and liver qi stagnation syndrome are enrolled. Patients will be randomly allocated to the QTG or placebo groups at a 1:1 ratio. QTG and placebo will be added to the modern guideline-directed medical therapy for 12 weeks and patients will be followed up for another 24 weeks. The primary outcome is the improvement of metabolic equivalents measured by CPET. The secondary outcomes are cumulative incidence of composite endpoint events, other indicators in CPET, changes in the Seattle Angina Questionnaire, traditional Chinese medicine syndrome scale, 12 items of Short Form Health Survey Questionnaire, Patient Health Questionnaire-9, and Generalized Anxiety Disorder-7, changes of ST-T segment in the electrocardiogram, improvement of left ventricular ejection fraction and left ventricular end-diastolic diameter in echocardiography. In addition, metabolomics analysis will be performed based on blood samples. Adverse events and safety evaluations will also be documented. A full analysis set, per protocol set, and safety analysis set will be conducted.

**Discussion:**

This clinical trial can enrich treatment options for CHD patients with low cardiorespiratory fitness and psychological imbalance, and it may also create a new situation for promoting the application of traditional Chinese medicine in cardiac rehabilitation.

**Clinical Trial Registration:** [http://www.chictr.org.cn], identifier: [ChiCTR2200058988].

## Introduction

Statistics from the American Heart Association revealed that there were approximately 244 million patients with ischemic heart disease (IHD) and 8.95 million deaths of IHD worldwide in 2020, with an age-standardized IHD mortality rate of 112/100,000 (1). Despite modern guideline-adjusted therapy, patients with stable coronary artery disease (SCAD) still have a high risk of recurrent cardiovascular events (2, 3). Ischemic heart disease remains the leading cause of death worldwide (4, 5). Urgent percutaneous coronary intervention (PCI) is crucial to save the life of patients with acute myocardial infarction. However, compared with conservative medication, the additional increase in life expectancy provided by coronary artery revascularization for SCAD patients is controversial (6, 7). Patients who receive optimal medical treatment still have high residual cardiovascular risk (8); meanwhile, they often complained of recurrent angina pectoris, anxiety, and depressive symptoms, declined exercise tolerance and quality of life, and impaired social function (9–11). The multiple benefits of exercise-based cardiac rehabilitation (CR) on cardiovascular outcomes have been well testified (12). Exercise tolerance refers to the maximum exercise capacity the body can achieve, and it is a strong and independent predictor for all-cause and cardiovascular-specific mortality (13). Cardiopulmonary exercise testing (CPET) can precisely evaluate exercise capacity and functional status (14). At present, drugs that can significantly enhance the exercise tolerance of cardiovascular patients are rare.

In traditional Chinese medicine (TCM), the core pathogenesis of SCAD is “deficiency in origin and excess in superficiality”, while anxiety and depression are associated with liver-qi stagnation. Qi deficiency can lead to blood stasis, and it can also result in qi stagnation. In other words, this is “excess resulting from deficiency”. Based on this theory, Qixue Tongzhi Granule (QTG) is transformed from Xuefu Zhuyu decoration by academician Chen Keji. QTG consists of *Astragali radix*, *Chuanxiong rhizoma*, *Paeoniae radix rubra*, *Corydalis rhizoma*, and *Aurantii fructus*, having the function of tonifying qi, promoting blood circulation, and regulating qi-flowing. During the 8th, 9th, and 10th Five-year National Science and Technology Attack Plan, academician Chen Keji's team conducted a series of clinical trials to evaluate the effect of optimized formulas of Xuefu Zhuyu decoration, such as Xuefu Zhuyu condensed pills, refined Xuefu capsule, Xiongshao capsule, on preventing restenosis after coronary intervention (15, 16). Declined exercise capacity and fatigue are common clinical manifestations in CHD patients. Evidence from the basic research shows that *Astragali radix* can increase exercise capacity and resist fatigue in mice (17, 18). Moreover, *Astragali radix* can lower cholesterol levels and improve self-reported vigor in older adults (19). Active ingredients extracted from *Paeoniae radix alba*, *Corydalis rhizoma*, *Aurantii fructus*, and *Astragali radix* have antidepressant effects in animal models (20–23).

Under the direction of disease integration with the syndrome theory, we aim to investigate the efficacy and safety of QTG in improving exercise capacity, alleviating angina pectoris, anxiety/depression symptoms, promoting the health-related quality of life, and reducing the risk of composite endpoints in subjects with SCAD. Until now, Chinese and Western medicines verified to be effective in improving aerobic capacity and prognosis of CHD are rare. This study may present a novel, promising treatment option for CHD patients with low cardiorespiratory fitness and psychological imbalance.

## Methods and materials

### Design and settings

This is a multicenter, prospective, randomized, double-blind, placebo-controlled, superiority trial. This trial is registered in the Chinese Clinical Trial Registry (No. ChiCTR 2200058988) and fully complies with the Declaration of Helsinki and Good Clinical Practice (GCP) Guidelines 2020. The protocol has been designed according to SPIRIT 2013 statement and SPIRIT 2013 explanation and elaboration (24, 25). The study was approved by the Research Ethics Committee of Xiyuan Hospital, China Academy of Chinese Medical Sciences (ID: 2021XLA090-3).

**Figure 1:**
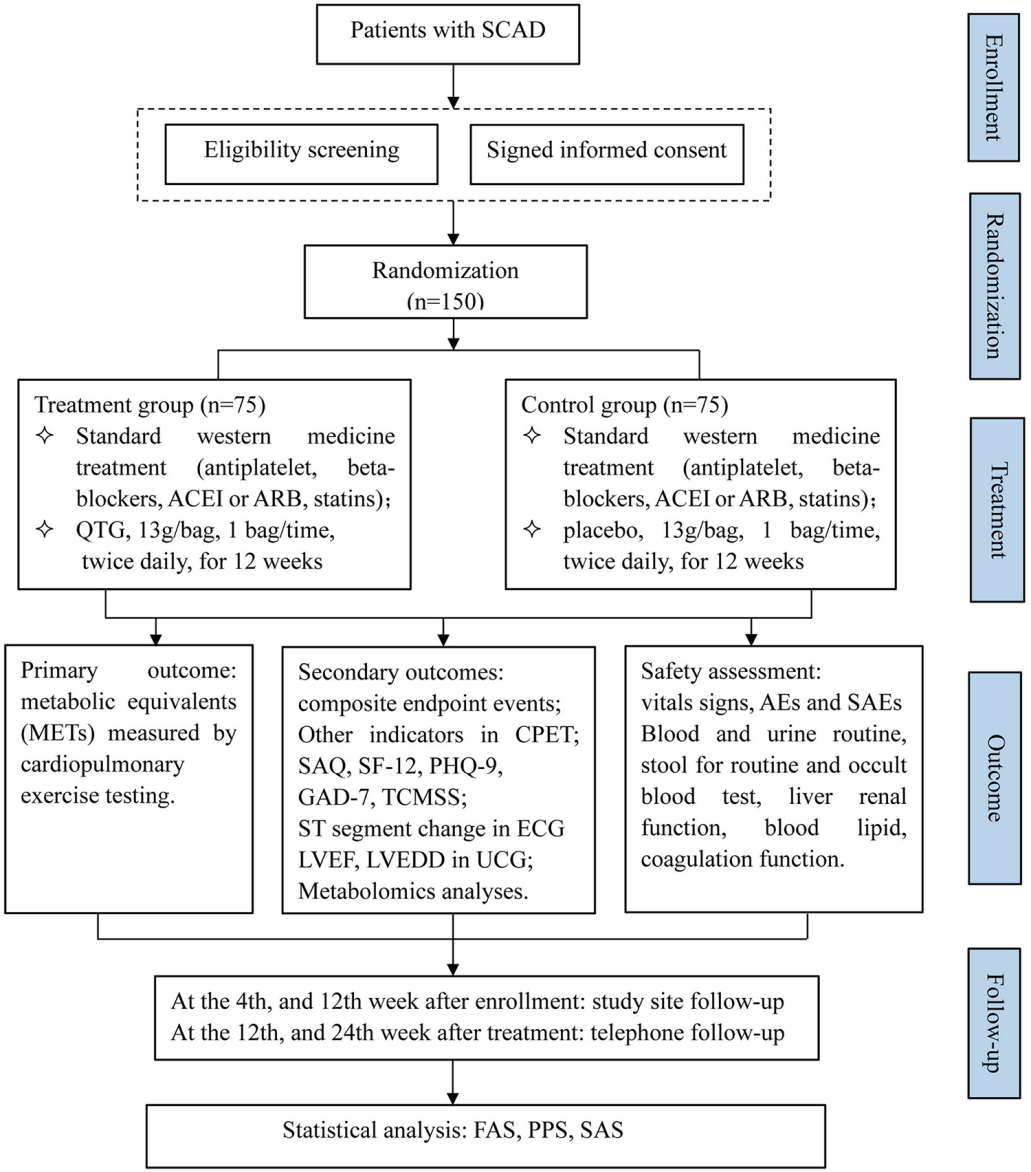
Flow chart of the study design. SCAD, stable coronary artery disease; ACEI, angiotensin-converting enzyme inhibitor; ARB, angiotensin receptor antagonist; QTG, Qixue Tongzhi Granule; CPET, cardiopulmonary exercise testing; SAQ, Seattle angina questionnaire; SF-12, 12 items of short form health survey questionnaire; PHQ-9, patient health questionnaire-9; GAD-7, generalized anxiety disorder-7; TCMSS, traditional Chinese medicine syndrome scale; ECG, electrocardiogram; LVEF, left ventricular ejection fraction; LVEDD, left ventricular end-diastolic diameter; UCG, echocardiography; AEs, adverse events; SAEs, severe adverse events; FAS, full analysis set; PPS, per protocol set; SAS, safety analysis set.

This multicenter trial will be conducted at four hospitals in mainland China, including Xiyuan Hospital, Guanganmen Hospital, Wangjing Hospital, and The First Affiliated Hospital of Shanxi University of Chinese Medicine. A total of 150 participants will be recruited. After the participants have provided written informed consent, they will be enrolled in the trial, which consists of a 12-week treatment period and a 24-week follow-up period. The schematic diagram of study procedures is illustrated in Figure 1.

### Study population

#### Diagnostic criteria

The diagnostic criteria of SCAD refer to the 2023 AHA/ACC/ACCP/ASPC/NLA/PCNA guideline for the management of patients with chronic coronary disease (26); The diagnosis of TCM syndrome elements of qi deficiency, blood stasis, and qi stagnation follows expert consensus on TCM diagnosis and treatment of stable angina pectoris of coronary heart disease (2018 edition) (China Association of Chinese Medicine).

#### Inclusion criteria

Patients eligible for the trial must comply with all of the following at randomization:

1) Coronary angiography or coronary computed tomographic angiography shows at least one major coronary artery luminal diameter stenosis ≥ 50%, a history of myocardial infarction, or being in a stable condition more than one month after PCI or coronary artery bypass grafting.
2) Left ventricular ejection fraction ≥ 40%.
3) Canadian Cardiovascular Society grading of angina pectoris is I-II.
4) TCM syndrome differentiation is in accord with qi deficiency blood stasis and liver qi stagnation. TCM syndrome diagnosis should have the primary symptoms and at least two accompanying symptoms. Primary symptoms: chest tightness and chest pain; Accompanied symptoms: palpitation, shortness of breath, spontaneous perspiration, fatigue, distension in the hypochondrium, abdominal fullness, heaving a deep sigh, impatience, and irritability.
5) Aged 18–75 years.
6) Meet low to medium risk stratification of CR and could take part in the CPET.
7) Provide written informed consent.

#### Exclusion criteria

Patients who meet any of the following criteria couldn't attend the study:

1) Acute myocardial infarction, unstable angina pectoris, underwent PCI or CABG within the past month.
2) Meet absolute and relative contraindications of CPET.
3) Patients who have taken botanical drugs for tonifying qi, promoting blood circulation, or regulating qi flowing, or have participated in other clinical trials during the past month.
4) Abnormal renal function with an estimated or calculated creatinine clearance < 60 ml/min.
5) Patients with severe liver disease or alanine aminotransferase, aspartate transaminase > 3 times the upper limit of normal.
6) New York Heart Association class Ⅳ or recurrent malignant arrhythmia.
7) Combining with chronic obstructive pulmonary disease, pulmonary infection, or respiratory failure.
8) Diabetic patients with random blood glucose ≥ 13.7 mmol/L or glycosylated hemoglobin ≥ 9.5%.
9) Pregnancy, preparing for pregnancy, and breastfeeding.
10) Patients have acute cerebrovascular disease, malignant tumor, serious hematopoietic system disease, or life expectancy of less than one year.
11) Patients with psychosis, severe anxiety, and depression or those who have taken anti-anxiety and depression drugs within the past month;
12) Patients are allergic to known ingredients of the study drug.

#### Criteria for withdrawal, dropout, removal

If the subjects occur severe organ dysfunction, drug allergic reaction, disease deterioration, or serious adverse events during the trial, it is necessary to stop participating in the trial according to the judgment of clinicians; participants can also withdraw from the trial voluntarily due to poor efficacy, intolerance of adverse reactions, and desire to try another treatment methods. Of course, participants may withdraw from the study for any reason at any time. Although the subjects do not explicitly propose to withdraw from the study, they no longer receive medications and examinations and are lost to follow-up. Undoubtedly, this is also withdrawn.

Dropout means that randomized patients prematurely discontinue the study and only complete some of the procedures specified in the trial. For patients who were lost to follow-up or withdraw, efforts should be made by telephone or letter to inquire about the reasons for dropping, investigate their medication history and the time of last taking medicine, and record the outcome measurements at the time of discontinuation. Investigators should try their best to keep subjects enrolled in the study and have the following clinical and laboratory evaluations performed.

Participants may be removed if he/she (1) violates inclusion criteria or meets the exclusion criteria, or receive the wrong treatment assignment; (2) has poor treatment compliance or the number of drugs used does not meet the requirements (<80% or >120%); (3) has incomplete data and no evaluable records after randomization; (4) has used drugs that may influence the efficacy and safety evaluation of the study drug. Removal cases should not be included in the efficacy analysis. However, the safety analysis should incorporate those who receive at least one treatment and have at least one safety record.

### Randomization, allocation concealment, and blinding

#### Random allocation

All patients who consent to participate and fulfill the inclusion criteria will be randomly assigned to the control or QTG group with a 1:1 allocation ratio. An independent statistician will generate the randomization sequence using center-stratified block randomization via SAS software (version 9.4). Treatment allocations will be put into opaque sealed envelopes sequentially numbered 1 to 150. A statistician will send the blind codes directly to the drug manufacturing room to label investigational products. Treatment allocation will not be disclosed until the primary endpoint is analyzed to ensure concealment. According to the clinical study program, the randomized code of drugs is the unique identification code of participants.

#### Blinding

After assigning interventions, participants, investigators, data collectors, outcome assessors, and data analysts will be blinded.

#### Unblinding and emergency unblinding

This study has second-level blinding. After all the research data are checked and locked, the principal investigator (PI) and statistician will conduct the first unblinding, revealing the groups (such as groups A and B) to which each random number belongs. After completing the statistical analysis and clinical trial summary report, the second unblinding will be performed at the closing meeting to uncover the actual drug (QTG or placebo) each group takes.

Emergency unblinding is permissible only in exceptional circumstances, such as severe adverse events suspected to be related to the investigational products when knowledge of the actual treatment is essential for further patient management. Once unblinded, the participant will withdraw from the study. Researchers should report to the PI and ethics committee immediately and report the reasons for unblinding within 24 h. Investigators must record all code breaks with reasons as they occur on the corresponding case report form (CRF) pages.

#### Sample size calculation

The sample size calculation is based on the superiority trial hypothesis test and is driven by the expected improvement in metabolic equivalents (METs). Referring to data from preliminary clinical trials (27, 28), we assume that the minimum difference in improvements of METs between groups over a 12-week treatment period would be 1.38, and the combined standard deviation would be 1.56. According to relevant literature and experts' advice, the superiority margin is 0.42. Considering a 20% dropout rate and referring to calculation results from PASS 15.0 software, 144 participants would achieve 90% power and a one-sided 2.5% significance level. For the convenience of randomization, a sample size of 150 is identified.

#### Interventions

Concerning standard Western medicine treatment during the trial, according to the guidelines for SCAD treatment (29), antiplatelet drugs, β-blocker, statins, and angiotensin-converting enzyme inhibitors/angiotensin receptor antagonists should be used. Hypoglycemic, hypotensive, and lipid-lowering drugs, as well as other chemical drugs, are permitted depending on the actual condition of the patients. Researchers should record concomitant medications truthfully and maintain dose stability during the trial.

#### Treatment group

The investigational products are produced by the TCM preparation room of Xiyuan Hospital. The composition of QTG includes *Astragali radix* 30 g*, Chuanxiong rhizoma* 10 g*, Paeoniae radix rubra* 10 g*, Corydalis rhizoma* 10 g*,* and *Aurantii fructus* 10 g (Table 1). The pharmacy department purchases and identifies all herbal decoction pieces in QTG. The above five Chinese herbal medicines are prepared in proportion and decocted twice with water, the first time five times water for 30 min, the second time three times water for 20 min, the decoction of two times is combined and filtered, then the filtrate is concentrated into a sugarless paste with a relative density of 1.15–1.25 (50°C). Take the above paste, and add the appropriate amount of dextrin, then QTG is produced using one-step pelletization technology. Granules are packaged into 13 g/bag. Usage and dosage: one bag/time, twice daily, taking medicine orally after mixing it with 150 ml hot water. The treatment course will last 12 weeks. Patients are asked to return unused drugs and drug packages to the investigators at each visit to assess medication adherence.

**Table 1:** The composition of Qixue Tongzhi Granule (intervention drug).

Chinese name	Scientific name	Latin name	Species name	Amount
Huang qi	*Astragalus membranaceus* (Fisch.) Bge.	*Astragali radix*	Leguminosae	30 g
Chuan xiong	*Ligusticum chuanxiong* Hort.	*Chuanxiong rhizoma*	Apiaceae	10 g
Chi shao	*Paeonia lactiflora* Pall.	*Paeoniae radix rubra*	Ranunculaceae	10 g
Yan hu suo	*Corydalis yanhusuo* W.T. Wang.	*Corydalis rhizoma*	Papaveraceae	10 g
Zhi qiao	*Citrus aurantium* L.	*Aurantii fructus*	Rutaceae	10 g

#### Control group

The main ingredients of the placebo granule are caramel and dextrin. In addition, we have added a 10% crude drug of QTG to the placebo granule to achieve smell, color, taste, and texture comparable to that of QTG. Per 13 g/bag of placebo contains 0.0459 g of caramel. The usage and dosage of placebo are the same as QTG.

#### Drugs combined and contraindicated in trial

Patients with sudden onset of angina pectoris can take nitroglycerin or Suxiao Jiuxin Pills under the tongue temporarily. However, they need to detail the date, duration, and alleviation. All newly added medications and treatments other than those originally prescribed drugs should be faithfully recorded in the CRF. During the study period, all other traditional Chinese medicine decoctions, oral or intravenous Chinese patent medicines, or TCM-appropriate technology (including acupuncture or cupping), trimetazidine, coenzyme Q10, and other drugs that may affect the clinical efficacy evaluation are prohibited; anxiolytics or antidepressants are prohibited as well.

#### Follow up

After enrollment, all participants will be followed up four times; the first two times at the 4th and 12th weeks after enrollment are study site follow-ups, while the later two times at the 12th and 24th weeks after treatment are telephone follow-ups.

#### Outcome

The details of items to be measured and time points for data collection are shown in Table 2.

**Table 2:** Trial schedule.

Study phase		Screening period	Intervention period (12 weeks)		Follow-up period (24 weeks)	
Visit		V1	V2	V3	V4	V5
		−3–0 days	Week 4 ± 2 days	Week 12 ± 3 days	Week 24 ± 5 days	Week 36 ± 7 days
Inclusion/exclusion criteria		×				
Informed consent		×				
Random allocation		×				
Baseline data collection	Demographic information	×				
	Medical history	×				
	Past medical history, allergies	×				
Safety evaluation	Vital signs	×	×	×		
	Blood, urine, and stool routines	×	×	×		
	Liver and renal function	×	×	×		
	Blood lipids	×	×	×		
	coagulation function	×	×	×		
	Adverse events		×	×	×	×
Outcome evaluation	CPET	×		×		
	Composite endpoint events		×	×	×	×
	Other indicators in CPET	×		×		
	SAQ, SF-12	×	×	×	×	×
	TCM syndrome scale	×	×	×	×	×
	PHQ-9, GAD-7	×	×	×	×	×
	echocardiography	×		×		
	electrocardiogram	×		×		
	Metabolomics analyses	×		×		
Drug distribution		×	×			
Recovery drug			×	×		
Record drug combination			×	×	×	×
Evaluate compliance				×		

CPET, cardiopulmonary exercise testing; SAQ, seattle angina questionnaire; SF-12, 12 items of short form health survey questionnaire; TCM, traditional Chinese medicine; PHQ-9, patient health questionnaire-9; GAD-7, generalized anxiety disorder-7.

#### The primary and secondary outcomes

The primary outcome is the improvement of METs in CPET. METs are an important indicator for evaluating aerobic metabolism and cardiorespiratory fitness. 1 MET equals 3.5 ml O_2_/kg/min. An increase in exercise capacity manifested by MET can bring great survival benefits. Small increases in cardiorespiratory fitness (e.g., 1–2 METs) are associated with considerably (10% to 30%) lower adverse cardiovascular event rates (30). CPET will be performed at baseline and the end of the 12-week treatment period. Changes from the baseline will be compared between groups.

The secondary outcomes are as follows:

1) Composite endpoint events include cardiovascular death, nonfatal myocardial infarction, revascularization, hospitalization for heart failure or unstable angina pectoris, stroke, and other thromboembolic complications. It will be recorded throughout the study. At the end of the 24-week follow-up period, the cumulative incidence of composite endpoint events will be compared between groups.
2) Other indicators in CPET: (a) peak oxygen uptake (PeakVO_2_), percentage of predicted PeakVO_2_, oxygen pulse (VO_2_/Heart rate, VO_2_/HR), oxygen uptake related to work rate (ΔVO_2_/ΔWR), Ventilation Aerobic Threshold (VAT). These indicators reflect exercise capacity and cardiac function. (b) The partial pressure of end-tidal carbon dioxide (PETCO_2_), ventilatory equivalent for carbon dioxide slope (VE/VCO_2_ slope). These parameters measure the body's ability to exchange gases. Comparisons between groups will be made on the 12th week after enrollment.
3) Seattle Angina Questionnaire (SAQ). SAQ is a valid self-administered scale that estimates five dimensions of health in patients with SCAD: physical limitation, anginal stability, anginal frequency, treatment satisfaction, and disease perception. SAQ will be measured at every visit. Changes from baseline in each dimension of SAQ will be compared between groups.
4) TCM syndrome scale (TCMSS). The TCMSS is composed of related symptoms and signs (chest pain, chest tightness, palpitation, shortness of breath, spontaneous sweating, fatigue, distension in the hypochondrium, abdominal distension, heave a deep sigh, impatience and irritability, tongue manifestation, and pulse condition). Scores are assigned according to the severity of items. Measurement will be performed at each visit. The efficacy evaluation criteria are (a). Markedly effective: Clinical symptoms and signs are significantly improved, and syndrome scores are reduced by ≥ 70%. (b) Effective: Clinical symptoms and signs have improved; syndrome scores have been reduced by ≥ 30% and <70%. (c) Invalid: no significant improvement in clinical symptoms and signs, or even aggravation, syndrome score reduction < 30%. (d) Aggravation: The clinical symptoms and signs were aggravated, and the syndrome score decreased < 0.
5) 12 items of Short Form Health Survey Questionnaire (SF-12), Patient Health Questionnaire-9 (PHQ-9), and Generalized Anxiety Disorder-7 (GAD-7). SF-12, PHQ-9, and GAD-7 will be measured at each visit to evaluate patients' quality of life and current emotions of anxiety and depression, respectively. Changes from the baseline will be compared between groups.
6) ST-T segment change in electrocardiogram, improvement of left ventricular ejection fraction (LVEF), as well as left ventricular end-diastolic diameter (LVEDD) in echocardiography will be compared between groups to assess the effect of QTG on myocardial blood supply and cardiac contractile functions at the 12th weeks after enrollment.
7) Metabolomics analyses: Blood samples from ten participants per group will be elected randomly for metabolomic analyses. Liquid chromatography-mass spectrometry (LC-MS) and gas chromatography-mass spectrometry (GC–MS) will be adopted to detect the chemical and biological fingerprints, filter effective components, and explore the underlying metabolic pathways and target molecules of QTG for treating SCAD. Concerning the collection of blood samples, participants should fast for at least 8 h. 5 ml blood will be drawn into heparin sodium anticoagulant tubes, centrifuged, and plasma stored in Eppendorf tubes at −80°C.

#### Safety assessment and adverse events report

This study will evaluate safety by monitoring adverse events (AEs), serious adverse events (SAEs), withdrawals, or treatment alterations due to AEs. In addition, laboratory tests (blood routine, urine analysis, stool for routine and occult blood test, liver and renal function, blood lipid, and blood coagulation function) and vital signs will be conducted to assess the safety of QTG. Safety evaluation will be performed at baseline, the fourth week during treatment, and at the end of treatment. All SAEs will be reported to the ethics committee. The severity of AEs will be graded using CTCAE version 5.0. When subjects suffer from trial-related personal injury, appropriate treatment measures will be taken timely to ensure their safety. Subject compensation claims will also be properly handled.

#### Data collection and management

A paper CRF is used for data collection, and the laboratory and inspection results should be accurately, completely, and normatively recorded on the CRF promptly. After the clinical trial, CRFs will be checked by the clinical monitor for data integrity and then handed over to the data administrator for entry. Electronic CRF in the electronic data capture (EDC) system is designed strictly adhere to CRF's research protocol and content. All data will be input electronically at Xiyuan Hospital when the last patient is followed up. Two groups of trained researchers will conduct independent double data entry to ensure accuracy. The database support data format, valid values, and range checks. Questions proposed by data administrators will be sent to investigators by monitors in the form of data query reports. Researchers will respond by checking the original material. Data entry clerks will update the database according to the reply contents on the query reports. Adverse events, medical diagnoses, concomitant medications, past medical history, etc., collected from the CRF will be coded in terms from the Medical Dictionary for Regulatory Activities (MedDRA). After the data cleaning, a blind review meeting will be held, and the database will be locked. All paper files related to the study data will be stored in numerical order and kept in locked cabinets. Access to the study data will be restricted. Participant files will be maintained in storage for five years after completing the study.

#### Data analysis

The statistical analysis will be conducted using SAS software (version 9.4). According to the “intention to treat” principle, the full analysis set (FAS) is defined as all participants randomized in the trial, with at least one experimental drug treatment history and at least one therapeutic session visit. Per protocol set (PPS), exclude participants found to be ineligible after randomization or deviate from the intervention or follow-up protocols. The safety analysis set will include all randomized patients who have completed at least one study visit. Finally, we will draw conclusions according to the analysis results of FAS. PPS will be used for sensitivity analysis.

For quantitative data meeting normal distribution, means with standard deviations will be calculated, otherwise, medians with quartile ranges will be shown. While for qualitative data, frequencies and percentages will be described. Comparison between groups will be performed by independent t-tests, or Wilcoxon rank-sum test for quantitative data, and the chi-square test or Fisher's exact test for qualitative data. Subgroup analyses will be conducted on those with PHQ-9 or GAD-7 scores ≥ 5 or < 5. Confounding factors that are difficult to control and unbalanced between groups will be used as covariates to analyze covariance to eliminate their influence on efficacy evaluation. *P* < 0.05 will be considered statistically significant. The last observation carried forward is supposed to estimate missing outcome data. Regarding safety, the number of AEs and SAEs will be categorized, and the incidence rate will be calculated. The data monitoring committee is not formally established because the trial has a short duration and known minimal risks. However, some form of data monitoring will undoubtedly be conducted by inspectors appointed by PI to ensure the accuracy and integrity of data.

#### Quality control

Inspectors assigned by the PI will visit branch centers three times a year during the trial to monitor the conduct of the clinical trial. The PI will arrange several clinical research coordinators to solve problems generated during the trial and strengthen contacts between trial centers. Before trial initiation, physicians, investigators, outcome assessors, and statisticians will be trained uniformly regarding the study objective, study protocol, informed consent, and relevant standard operating procedure. An independent clinical event committee represented by academician Chen Keji will be set to distinguish the major adverse cardiovascular events (MACEs).

Measures taken to reduce bias: All operators in these four research centers will receive training correlated with the standard operation procedures, cautions, and termination indicators of CPET and finally undergo a consistency evaluation. A symptom-restricted scheme with a ramp power escalation pattern will be adopted. The work rate increasing rate of CPET will be calculated according to the participant's age, sex, height, and weight (31). For the same subject, the work rate increasing rate will stay the same before and after the trial. In addition, CPET outcomes are easily influenced by the examination time and subjects' physical and emotional status. We demand that the examination time should be kept as consistent as possible for the same person. Participants are required not to perform high-intensity exercise 1 day before testing. Daily exercise intensity can also incur bias to METs, so participants are asked to keep similar daily exercise habits to before. Patients will be provided a diary card to record medication status and uncomfortable symptoms. Two Chinese medicine experts will blindly evaluate the TCM syndrome diagnosis of every participant.

Plans to promote participant retention and complete follow-up. Send text messages or give a ring to patients before the follow-up visit, reminding them of the upcoming data collection.

## Discussion

Atherosclerotic cardiovascular diseases are the leading cause of global mortality and a major contributor to disability. Continuing rising healthcare costs have caused substantial financial burdens to societies (32). Implementing effective prevention and treatment strategies is vital to reduce morbidity and mortality in CHD. The popularization of CR has shed light on the tough problem (33). Combined treatment of traditional Chinese medicine and Western medicine has been considered an effective CR mode. This trial is designed as a multicenter, randomized, double-blind, placebo-controlled study aiming to evaluate the effect of QTG on exercise tolerance, angina pectoris, anxiety/depressive symptoms, and prognosis in patients with CHD.

A retrospective cohort study of community-based exercise rehabilitation indicated that higher baseline submaximal cardiorespiratory fitness was associated with a reduced risk of all-cause mortality over 14 years in adults with CHD, and improved fitness was linked with significant risk reduction for the least fit (34). A study examining the prognostic value of exercise capacity in patients with nonvascularized and revascularized coronary artery disease manifested that exercise capacity was a strong predictor of mortality, MI, and downstream revascularizations. Furthermore, patients with similar exercise capacities had an equivalent mortality risk, irrespective of baseline revascularization status (35). CPET is the optimal instrument to measure patients' cardiopulmonary endurance noninvasively. To our knowledge, evidence regarding evaluating the effect of botanical drug products on the cardiopulmonary fitness of CHD patients is rare. So this trial has great significance in enriching alternative treatment therapies for CHD. The design and implementation of the study protocol completely conform to relevant international statements. We also take a series of measures to control quality throughout the study.

There also exist some limitations to this study. First, a 12-week therapeutic session and a 24-week follow-up visit are set. The relatively short duration of follow-up may be difficult to observe the occurrence of MACEs. However, previous studies have demonstrated that higher exercise tolerance is a good surrogate marker for long-term prognosis (13, 35, 36), so this trial may infer some guidance significance in Chinese herbal medicine improving the prognosis of SCAD. Second, the manufacturers of CPET equipment at four centers are not in full accordance with each other, so this may infer some measurement bias. While we will conduct unified training for outcome assessors concerning the operation scheme of CPET before trial initiation.

## Conclusion

This randomized, placebo-controlled trial evaluates the efficacy and safety of Qixue Tongzhi Granule in improving the exercise capacity and long-term prognosis of patients with SCAD, which may create a new situation for promoting the application of traditional Chinese medicine in cardiac rehabilitation.
